# New Mode of Reducing a Dislocation of the Lower Jaw

**Published:** 1850-10

**Authors:** 


### ARTICLE XIX.

### *New Mode of Reducing a Dislocation of the Lower Jaw.*

It is generally believed that in luxations of the lower jaw, the condyle lies in front of the transverse root of the zygoma, and is there held down either by muscular contraction, or by the resistance of the zygoma. This theory of J. L. Petit, Boyer, Sir A. Cooper, &c., has lately found an opponent in the person of M. Malgaigne; and M. Nélaton has recently brought forward some facts which confirms M. Malgaigne's opinion. According to these surgeons, the persistence of the displacement is caused by the contact existing between the coronoid process and the os malæ. Thus, it is sufficient, in order to reduce, to place the two thumbs on the coronoid processes after the patient has opened his mouth, and without taking hold of the jaw, or taking any fulcrum, to press them backwards, in order to make the condyles glide into their place. Another mode of proceeding, is to place one's self behind the patient, to take a fulcrum on the nape of the neck with the two thumbs, and place the fingers on the coronoid processes. The patient is then desired to open his mouth, and the surgeon makes a gentle pressure from before backwards on the coronoid process. Nothing more is required to bring the joint to a normal state. The method consists, then, merely in relaxing the muscles by opening the mouth, and in pressing moderately on the coronoid process to push it backwards, and a little downwards.—*Review Medico-Chirurgicale.*

